# ZnFe_2_O_4_ Nanotapers: Slag Assistant-Growth and Enhanced Photoelectrochemical Efficiency

**DOI:** 10.1186/s11671-017-1938-7

**Published:** 2017-03-23

**Authors:** Xuefeng She, Zhuo Zhang

**Affiliations:** 1 State Key Laboratory of Advanced Metallurgy, University of Science and Technology of Beijing (USTB), 100083 Beijing, People’s Republic of China; 2 Surface Chemistry Laboratory of Electronic Materials, Department of Chemical Engineering, Pohang University of Science and Technology (POSTECH), Pohang, 790-784 Korea

**Keywords:** ZnFe_2_O_4_, Rare-earth oxide slag, Photoelectrochemical, Hetero-nanostructure

## Abstract

In this study, ZnFe_2_O_4_ (ZFO) nanotapers are fabricated on the ZnO nanorods (NRs) by recycling rare-earth oxide (REO) slag as the iron source, which thereby exhibits dramatically enhanced photoelectrochemical (PEC) efficiency. Our studies demonstrate that the electron-hole separation and charge migration can be facilitated by the cascade band alignment of ZFO and ZnO and the branched nanotaper structures. Not only the iron source, the slag particles can also act as the passivation layers, leading to improved electron lifetime and significant PEC enhancement. The current study presents a novel REO-slag-modified PEC anode for high-efficiency PEC devices and offers a possibility of recycling industrial waste for renewable energy generation.

## Background

In the 21st century, the earth encounters unprecedented crises that demand urgent solutions to the issues concerning renewable energy and treatment of industrial waste. For the first issue, the skyrocketing demands for green energy have boosted the research of solar-energy conversion in the world. As a particularly promising technology in this regard, photoelectrochemical (PEC) processes enables a direct and efficient use of sunlight for hydrogen production [1–5]. In a typical PEC reaction, electron-hole pairs are generated from a semiconductor nanomaterial when it absorbs photons possessing higher energy than its band gap [6–8]. To date, many materials, such as CdSe [9–11], CdS [12, 13], and BiVO_4_ [14–16], have been identified as the promising PEC anode materials due to their relatively narrow band gaps. However, these materials are chemically unstable, which can gradually dissolve in strong basic and acidic solutions. Besides, the toxicity of Cd as well as the scarcity of Se and Bi in the earth also limit their applications. Comparatively, ZnFe_2_O_4_ (ZFO) is particularly suited as a material for PEC anode because it is abundant, nontoxic, and chemically stable in basic media [17]. Moreover, the band gap of about 1.9 eV enables it to utilize a large fraction of the solar spectrum [18]. As a result, various ZFO nanostructures with different morphologies have been designed, which have proven to exhibit highly efficient PEC performances. For example, Hou et al. reported that the PEC activity of the anode could be highly enhanced after the modification with ZFO/TiO_2_ composite nanotube arrays via an electrochemical method [19]; McDonald et al. also demonstrated that the Fe_2_O_3_/ZFO composite electrode showed a significantly enhanced photocurrent response compared to the bare Fe_2_O_3_ electrode [20]. All the previous results have suggested that ZFO is a promising material for PEC anode.

As for the second issue concerning the industrial waste, it is well known that in addition to the harvesting of renewable energies, the treatment or recycling of industrial wastes is also an urgent global challenge. As a typical industrial waste, metallurgical slag materials are plentifully available and broadly distributed. Generally, slag is a glass-like product derived from its raw ore, which usually consists of a consolidated mixture of various compounds, mostly CaO, SiO_2_, FeO, and other materials [21]. Most slag is used for fabricating cement. A special case is that the iron ore in China’s Inner Mongolia is commonly mixed with a large amount of rare-earth (RE) elements, such as Ce and La, making the slag rich in rare-earth oxides (REO) [22]. Table 1 shows that the major ingredients of REO slag are CaF_2_, SiO_2_, CaO, and REO, and the REOs are indicated with italics. Here, pure RE materials can be extracted from REO slags and have irreplaceable applications, however, most REO slags are either wasted or inefficiently utilized with minimal economic value produced. In our previous study, CaO and La_2_O_3_ in REO can effectively prevent the hydrolysis of the sacrificial reagent in electrolyte, leading to enhanced PEC efficiency [23]. Considering the promising application of ZFO to the PEC processes and the second issue mentioned above, great economic and social benefits could be derived from the fabrication of ZFO via recycling the REO slag.

**Table 1:** Composition of rare earth slag (%, mass fraction) [23]

CaF_2_	SiO_2_	CaO	Ce_2_O_3_	La_2_O_3_	P_2_O_5_	BaO	FeO	Rb_2_O	Dy_2_O_3_
32.25	25.71	15.82	4.73	2.64	2.31	2.03	1.64	0.93	0.73

In this study, the materials zinc and iron for fabricating ZFO are extracted from ZnO NRs and REO slag, respectively. The synthesis process can be divided into following steps: at first, the REO powder is mixed with the diluted solution of sulfuric acid (H_2_SO_4_) to form the REO suspension, during which Fe^2+^ ions are extracted from REO and dissolved in the suspension. Subsequently, NaOH solution is added to the REO suspension gradually until the pH value reaches about 7. Thirdly, the ZnO NRs are fabricated via hydrothermal method on the surface of F-doped SnO_2_ (FTO) glass, which are then dipped into the REO suspension. Under illumination, the electrons and holes are separated at the surface of ZnO, leading to the slight dissolution of ZnO and oxidization of Fe^2+^ to Fe^3+^. Therefore, FeO, CaO, and La_2_O_3_ are the three important materials for our hetero-nanostructures in Table 1. FeO could serve as the iron source for the fabrication of ZFO. CaO and La_2_O_3_ could increase the concentration of OH^-^ in PEC electrolyte, leading to depressed hydrolysis of S^2-^ ions and enhanced PEC efficiency. As a result, a porous compound structure containing Zn^2+^, Fe^3+^, and REO is formed on the surface of ZnO NRs. After annealing in the atmosphere, the ZFO NRs wrapped with REO layers are constructed on the surface of ZnO NRs, which is referred to as REO/ZFO NRs. The synthesis process is shown in the *Experimental Section* below, and the synthesis mechanism is discussed here: The fabricated REO/ZFO NRs demonstrate an enhanced PEC efficiency and are based on an economic and eco-friendly fabrication strategy. This synthetic strategy might be of great significance to improving the design and fabrication of future PEC iron-based photoanodes and also creating a promising prospect concerning the economic development of REO slag.

## Methods

### Fabrication

(1) *ZnO nanorods (NRs):* arrays of ZnO NRs were grown on FTO glass using a seed-assisted hydrothermal method. First, the ZnO seed layer was deposited uniformly on the native FTO glass substrate with the aid of radio frequency (RF) magnetron sputtering. The seeded FTO glass was then dipped into a mixed electrolyte that consists of hexamethylenetetramine (Sigma-Aldrich, HMTA, 20 mM) and zinc nitrate hexahydrate [Sigma-Aldrich, Zn(NO_3_)_2_°6H_2_O, 20 mM]. The deposition process typically lasted 12 h at 90 °C. (2) *Rare earth oxide (REO) powder*: first, cylindrical green briquettes with a uniform diameter of 25 mm and a height of 15 mm were formed by pressing 25 g of “Rare earth Bayan Obo complex iron ore” (simplified as RE ore), 12.71 g of coal and 7.8 pct of water (7.8 mL water mixed with 100.0 g RE ore) into a cylindrical mold for 1 min and dried at 378 K for 4 h in the oven. Dry briquettes were placed into a graphite plate and were heated at 1673 K (1400 °C) ±5 K for 12 h. Finally, the samples were rapidly removed from the furnace and subsequently cooled to ambient temperature. (3) *REO/ZFO NRs:* first, 20 g of REO powder is mixed into 100-mL-diluted solution of sulfuric acid (H_2_SO_4_) with concentration of 70%. At this step, Fe^2+^ ions are extracted from REO and are dissolved in the suspension. Second, 0.5-M NaOH solution is dropped gradually with stirring until the pH value about 7. Third, the ZnO NRs are dipped into the REO suspension. Fourth, the REO suspension with ZnO NRs is irradiated by white light (solar simulator) for 2 h. After that, the color of ZnO NRs is changed from white to orange. Next, after cleaning and drying, the ZnO NRs are annealed in the atmosphere at 500 °C for half an hour. Finally, the ZFO NRs wrapped with REO layer are constructed on the surface of ZnO NRs.

### Characterization

The morphologies of the prepared nanostructures were confirmed using a field-emission scanning electron microscope (FE-SEM, XL30S, Philips) operated with a 5.0 kV beam energy and a high-resolution scanning transmission electron microscope (HR-STEM; JEM-2200FS with Image Cs-corrector; JEOL) operated with a 200 kV beam energy. The spectra of X-ray diffractions (XRD) were measured in the range of 15–60° with a scan rate of 4°min^−1^ by using the diffractometer (D/MAX-2500, Rigaku) with Cu Kα radiation (40 kV, 100 mA).

### Measurements

(1) The optical absorbance of the samples was analyzed using a UV2501PC (Shimadzu) spectrometer with an ISR-2200 integrating sphere attachment for diffuse reflection measurements. (2) X-ray photoelectron scanning microscopy (XPS) was performed using an ESCALAB250 instrument (VG Scientific Company, USA) with Al Kα radiation as the excitation source. (3) *IPCE tests:* incident photon-to-current efficiency (IPCE) was measured using a 300-W Xe lamp (66 905, Oriel Instruments) with a monochromator (74-004, Oriel Cornerstone 130 1/8 m) of the wavelength from 300 to 700 nm at intervals of 20 nm. (4) Photocurrent-voltage (I–V) measurements, EIS, and electron lifetime test were performed using a typical three-electrode potentiostat system (potentiostat/galvanostat, model 263A, EG&G Princeton Applied Research) with a Pt counter electrode and a saturated calomel reference electrode (SCE). The electrolyte was an aqueous solution of 0.25 M Na_2_S and 0.35 M Na_2_SO_3_, through which nitrogen was bubbled. The working electrode was illuminated from the front with a solar simulator (AM 1.5 G filtered, 100 mW/cm^2^, 91160, Oriel).

## Results and Discussion

### Morphologies and Growth Mechanism

**Fig. 1:**
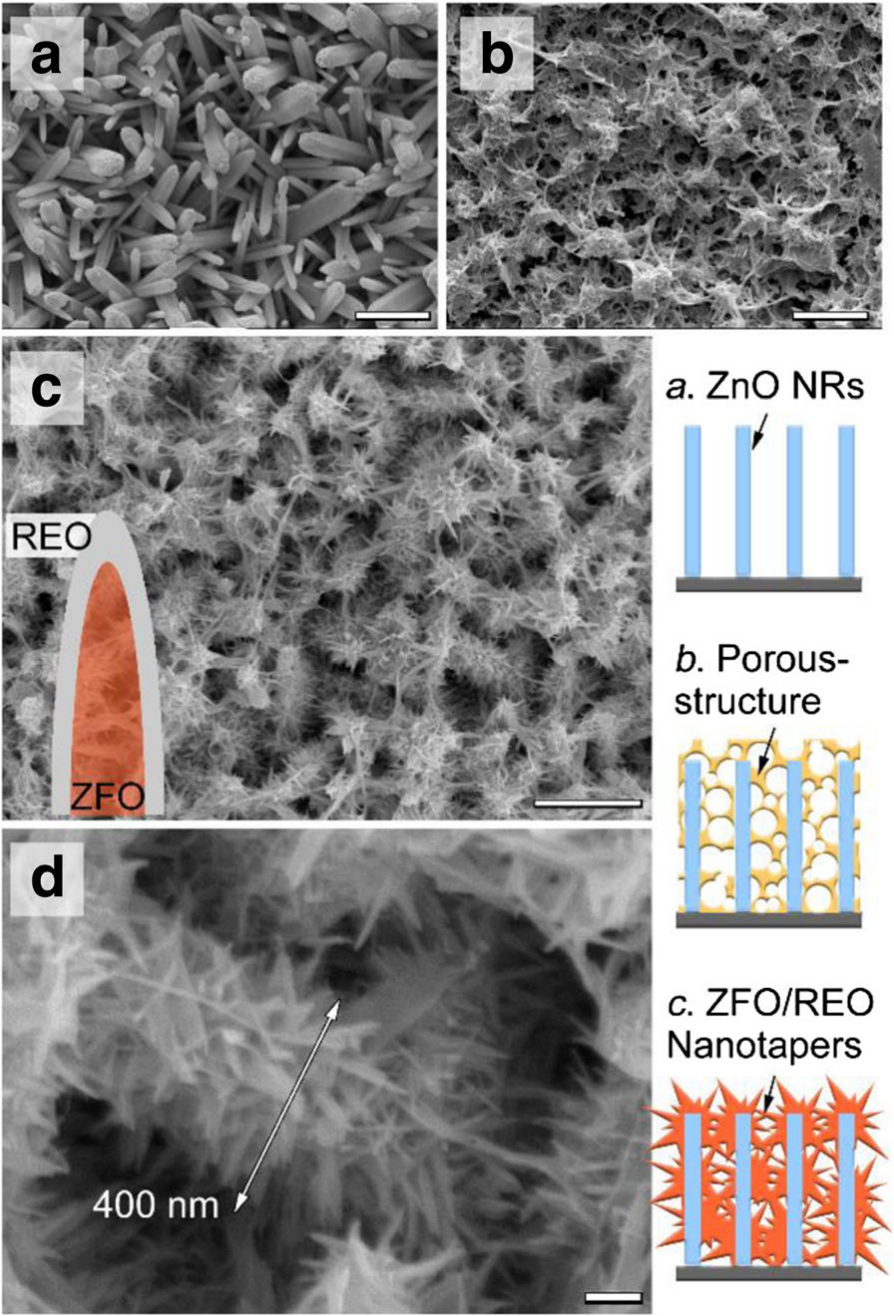
(**a**–**c**) SEM images of the bare ZnO NRs (**a**), ZnO NRs covered with the Fe^2+^/REO porous compound (**b**), and REO/ZFO nanotapers (**c**); (**d**) enlarged view taken from Fig. 1c. The *scale bars* are 1 μm in Fig. 1a–c and 100 nm in Fig. 1d. The sketches of these three structures are shown at the *lower right corner*

**Fig. 2:**
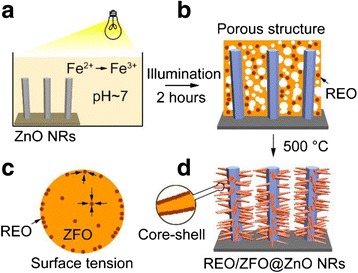
(**a**–**d**) Sketch depicting the key steps of fabricating the ZnO NRs covered with REO/ZFO nanotapers

SEM observations of the bare ZnO NRs and the ZnO NRs fully covered with ZFO/REO nanotapers are presented in Fig. 1. It can be seen from Fig. 1a that an array of ZnO NRs is distributed with high density on the FTO glass. After dipped into REO suspension and irradiated with white light, a porous structure is constructed on the ZnO NRs (Fig. 1b). After annealing, Fig. 1c demonstrates that the ZnO NRs are fully covered with ZFO-REO core-shell nanotapers. The enlarged view in Fig. 1c shows the the diameter of taper-covered ZnO NRs is about 400 nm. The lower right schematic diagram in Fig. 1 illustrates the evolving process, whose chemical mechanism is investigated here: at first, when REO powder is mixed into 70% H_2_SO_4_ solution, the Fe^2+^ ions are extracted from REO and dissolved in the REO suspension. Subsequently, NaOH solution is gradually added to the mixture until its pH value reaches about 7. Thirdly, ZnO NRs are dipped into REO suspension and irradiated by the white light (Fig. 2a). Under the illumination, the photo-generated electrons and holes could leave from the surface of ZnO [24]. As a result, the electrons reduce the H^+^ ions in the water and give rise to H_2_ gas, while the holes oxidize the Fe^2+^ ions in contact with ZnO to Fe^3+^. With the bubbling of H_2_, the concentration of OH^-^ increases in the suspension. As a result, the Fe^3+^ could react with OH^-^. The chemical reactions are listed below:

1 $$ 2{\mathrm{H}}^{+} + 2\mathrm{e}\ \left(\mathrm{electron}\right)\to {\mathrm{H}}_2\uparrow \left(\mathrm{gas}\right), $$

2 $$ {\mathrm{Fe}}^{2+}+\mathrm{hole}\to {\mathrm{Fe}}^{3+}, $$

3 $$ {\mathrm{Fe}}^{3+}+3{\mathrm{OH}}^{\hbox{-}}\to \mathrm{F}\mathrm{e}{\left(\mathrm{OH}\right)}_3\downarrow, $$

After washing and drying, the deposited compound presents a porous structure, containing Fe(OH)_3_ and REO precipitates on the ZnO NRs (Fig. 2b). Finally, the porous structure is transformed into ZFO nanotapers after annealing in the atmosphere at 500 °C (Fig. 2d). The ZFO nanotapers are wrapped with a layer of REO and erected on the ZnO NRs. The chemical reactions are:

4 $$ 2\mathrm{F}\mathrm{e}{\left(\mathrm{OH}\right)}_3\to {\mathrm{Fe}}_2{\mathrm{O}}_3+3{\mathrm{H}}_2\mathrm{O}, $$

5 $$ \mathrm{Z}\mathrm{n}\mathrm{O}+{\mathrm{Fe}}_2{\mathrm{O}}_3\to {\mathrm{ZnFe}}_2{\mathrm{O}}_4, $$

**Fig. 3:**
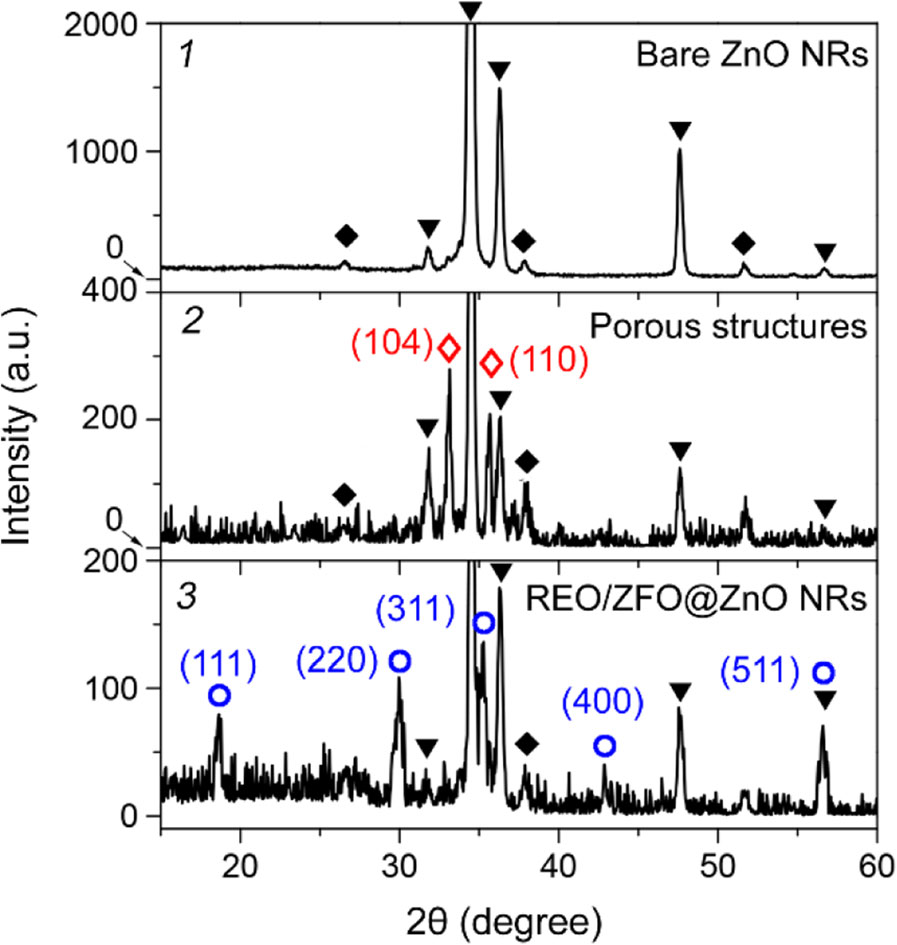
XRD spectra of the bare ZnO NRs (*1*), porous structure (*2*), and REO/ZFO@ZnO NRs (*3*). The diffraction peaks of ZnO, FTO, Fe_2_O_3_, and ZFO are marked by *black inverted triangle*, *black diamond suit*, *red lozenge*, and *blue circle*, respectively

**Fig. 4:**
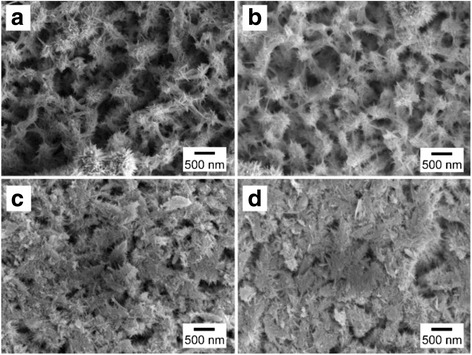
(**a**–**d**) SEM images of the ZnO NRs covered with REO/ZFO nanotapers prepared with different irradiated durations. **a** 1 h, **b** 2 h, **c** 3 h, and **d** 4 h

Here, the temperatures of reactions (4) and (5) should be higher than 75 and 450 °C [25], respectively. One thing should be noticed that the melting point of REO particles is higher than 1000 °C, while that of ZFO nanostructures is reported lower than 700 °C. As a result, during the formation of ZFO at 500 °C in our case, the REO particles will migrate to the surface of ZFO structure due to surface tension (Fig. 2c) [26], leading to the construction of ZFO-REO core-shell nanotapers (Fig. 2d). It should be noticed that, unlike Fe(OH)_3_, Ca(OH)_2_ precipitation cannot be formed when large amounts of calcium ions Ca^2+^ meet the OH^-^. Because Fe(OH)_3_ cannot dissolve in water, while Ca(OH)_2_ is slightly soluble. To further characterize the structures, XRD measurements on the bare ZnO NRs, porous structures, and REO are carried out. Figure 3(1) exhibits that only ZnO with hexagonal wurtzite structure and FTO can be observed on bare ZnO NRs. For porous structure shown in Fig. 3(2), the (104) and (110) peaks of Fe_2_O_3_ are present due to the reaction (4) mentioned above [27]. After annealing, the diffraction peaks of (111), (220), (311), (400), and (511) planes of ZFO are confirmed in Fig. 3(3), while those peaks of Fe_2_O_3_ are vanished [28]. It demonstrates that the Fe_2_O_3_ is fully changed as ZFO. Our studies also demonstrate that the densities of REO/ZFO nanotapers grown on ZnO NRs could be controlled. SEM images shown in Fig. 4a–d reveal that the density of REO/ZFO nanotapers is increased with the irradiated duration. Because equations (2) and (3) have confirmed that the amount of Fe(OH)_3_ could increase with the light irradiation.

**Fig. 5:**
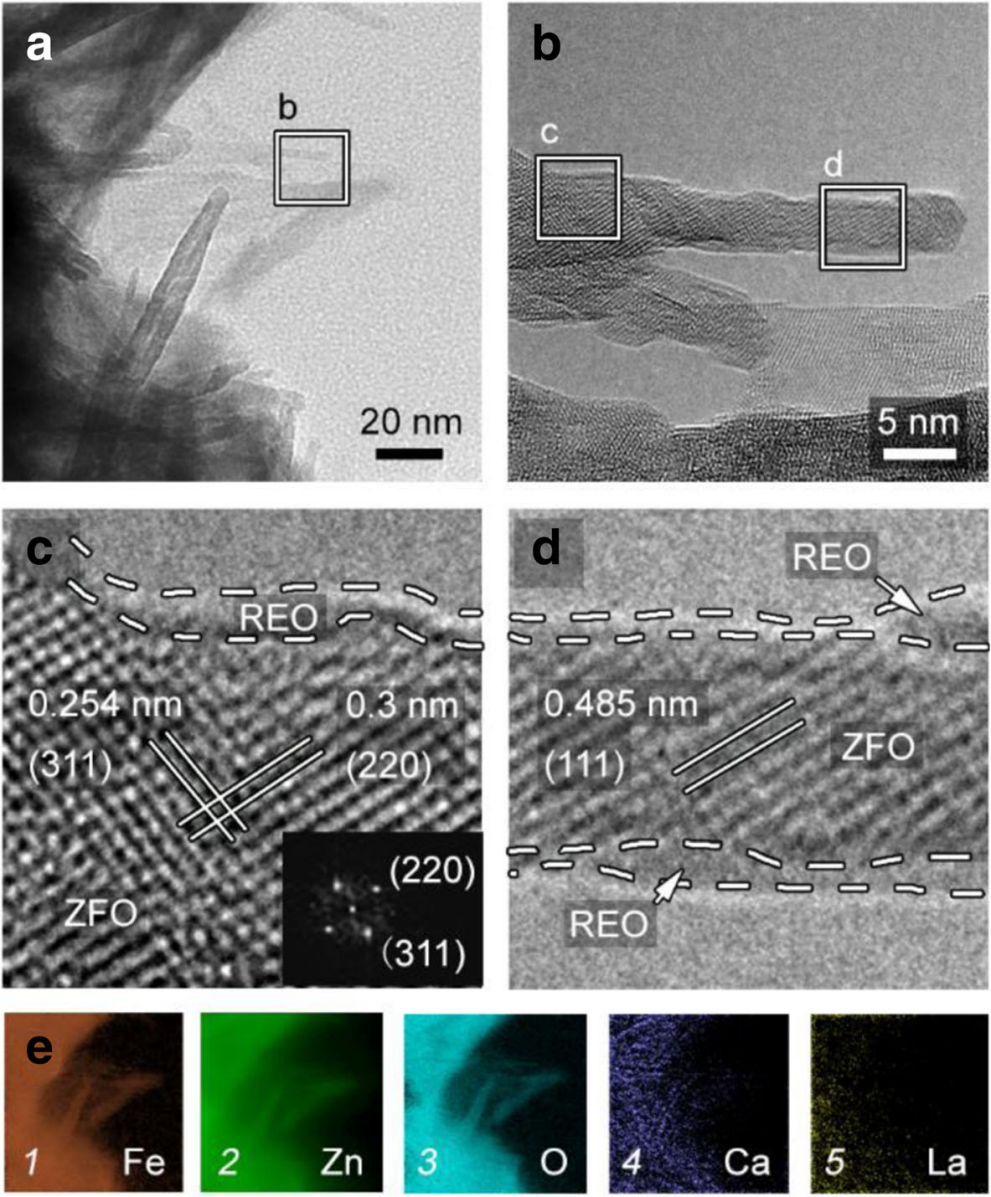
**a** TEM image of the REO/ZFO nanotapers. **b** Enlarged view taken from Fig. 3a. **c**, **d** HRTEM images taken from boxed regions in Fig. 3b. The inset is the FFT pattern. **e** Element mappings of Fe (*1*), Zn (*2*), O (*3*), Ca (*4*), and La (*5*) observed in Fig. 3a

Next, TEM observation is performed to clearly present the crystal properties of the REO/ZFO nanotapers. Figure 5a shows that the diameters of the nanotapers are about 5 ~ 20 nm. A close-up view of the boxed region in Fig. 5b is shown in Fig. 5c, which illustrates the HRTEM images. They indicate that the REO/ZFO nanotapers are well-crystalline and core-shell structures whose core is ZFO. The (311), (220), and (111) planes of ZFO are confirmed by measuring the distances between crystal planes [28]. The fast Fourier transform (FFT) pattern in the inset of Fig. 5c also shows the highly crystalline structure of ZFO. The REO layer tightly covers the ZFO as a shell and has a thickness of about 0.5 nm. The EELS element mappings in Fig. 5e 1–3 demonstrate that Fe, Zn, and O are evenly and precisely located at their respective positions. In our previous study, elements Ca and La in REO can efficiently enhance the PEC performance by preventing the hydrolysis of sacrificial chemicals, such as SO_3_^2−^, in PEC electrolyte. Hence, the mappings of Ca and La are also exhibited in Fig. 5e 4–5.

**Fig. 6:**
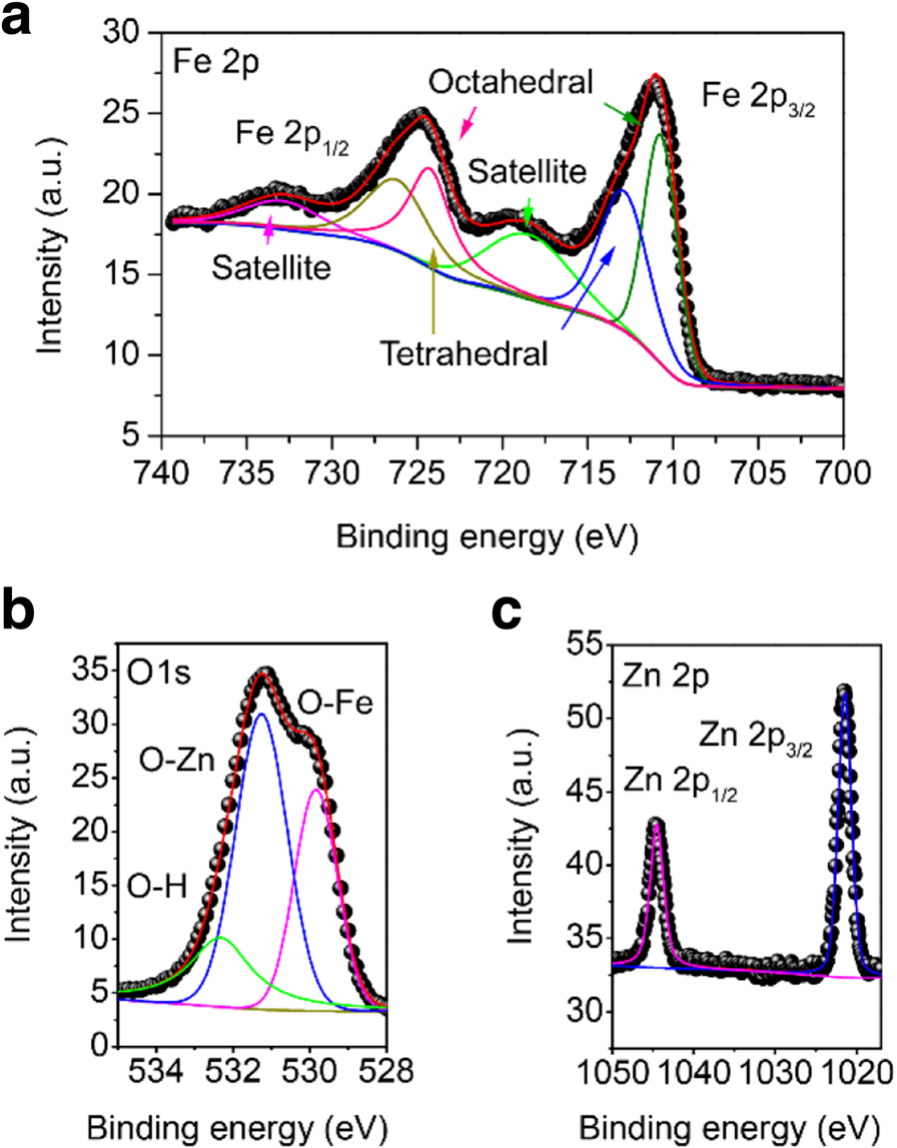
XPS spectra of Fe 2p (**a**), O1s (**b**), and Zn 2p (**c**) obtained from ZnO NRs covered with REO/ZFO nanotapers

To further study the chemical binding states of the synthesized materials, X-ray photoelectron spectroscopy (XPS) analysis is carried out. For REO/ZFO nanotapers, Fe 2p, O 1s and Zn 2p are collected in Fig. 6a–c, respectively. Via deconvolution of the XPS peak, it can be seen from Fig. 6a that the Fe 2p spectra consist of two groups of sub-peaks due to spin-orbit coupling at the 2p_1/2_ and 2p_3/2_ states. Each group possesses three sub-peaks corresponding to octahedral structure, tetrahedral structure, and satellite peak, respectively [29, 30]. Figure 6b demonstrates that the O 1s spectra consist of three sub-peaks due to spin-orbit coupling at 529.5, 531.1, and 533.2 eV, corresponding to the chemical bonding of Fe-O, Zn-O [31], and O-H, respectively. The Zn 2p spectra also have two sub-peaks at 1022 and 1047 eV, corresponding to 2p_1/2_ and 2p_3/2_ states, respectively [31]. The XPS result confirms that the nanotapers covering ZnO NRs are derived from the highly crystalline REO/ZFO core-shell materials and can serve as an ideal candidate for PEC anode.

### PEC Performances

**Fig. 7:**
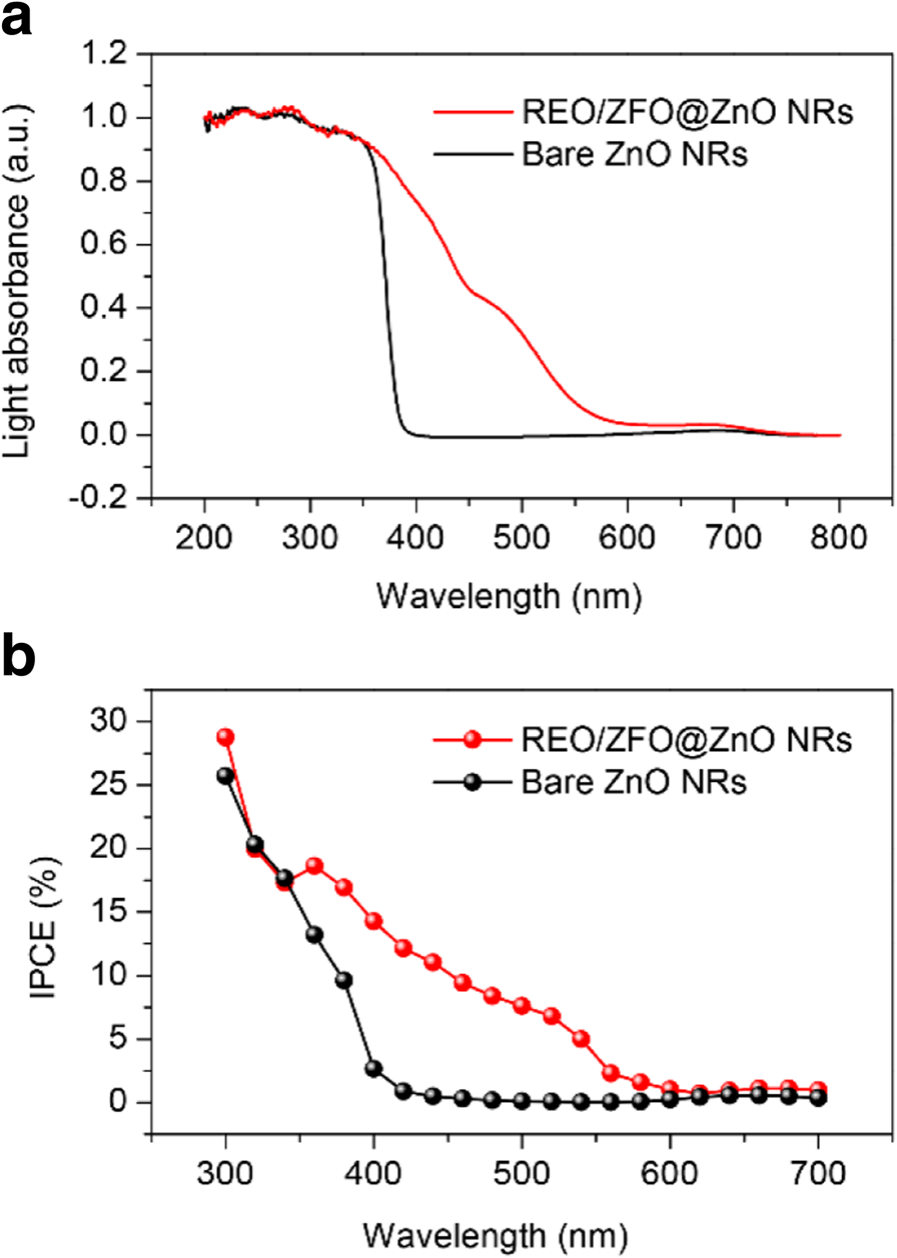
Light absorbance (**a**) and IPCE curves (**b**) of the REO/ZFO@ZnO NRs (*red*) and bare ZnO NRs (*black*) as functions of wavelength

The light absorptions and photon-to-electron conversion efficiency (IPCE) of the REO/ZFO nanotapers under the illumination with different wavelengths are studied to evaluate their contributions to PEC enhancement. The light absorbances of the bare ZnO NRs and the ZnO NRs covered with REO/ZFO nanotapers are selected and compared in Fig. 7a. Here, the ZnO NRs covered with REO/ZFO nanotapers are referred to as REO/ZFO@ZnO NRs in the figure. Bare ZnO NRs have enhanced light absorption existing solely in UV region with the wavelength less than 400 nm, while the light absorption of the REO/ZFO@ZnO NRs is enhanced from UV to visible light region. The IPCE spectra of the bare ZnO NRs (black) and REO/ZFO@ZnO NRs (red) are collected and compared in Fig. 7b as the electrodes at 0 V versus Ag/AgCl. The IPCE value at a given wavelength can be calculated via the following equation [32]:

6 $$ \mathrm{IPCE}\%=\frac{I_{sc}\left(\mathrm{A}\right)}{P\left(\mathrm{W}\right)}\times \frac{1240}{\lambda \left(\mathrm{nm}\right)}\times 100, $$

where *I*_sc_ denotes the measured photocurrent, *P* denotes the power of the incident light at a specific wavelength, and λ denotes the wavelength of the incident light. Figure 7b shows that, similar with the light absorbance, the IPCE curve of bare ZnO NRs shows enhancement only in the UV region due to the elevated UV photo-conversion of ZnO, while REO/ZFO@ZnO NRs exhibit considerable IPCE efficiency in visible light region. For both light absorption and IPCE curves, the visible photo-conversion of ZFO ascribes to its narrow band gap of 1.9 eV.

**Fig. 8:**
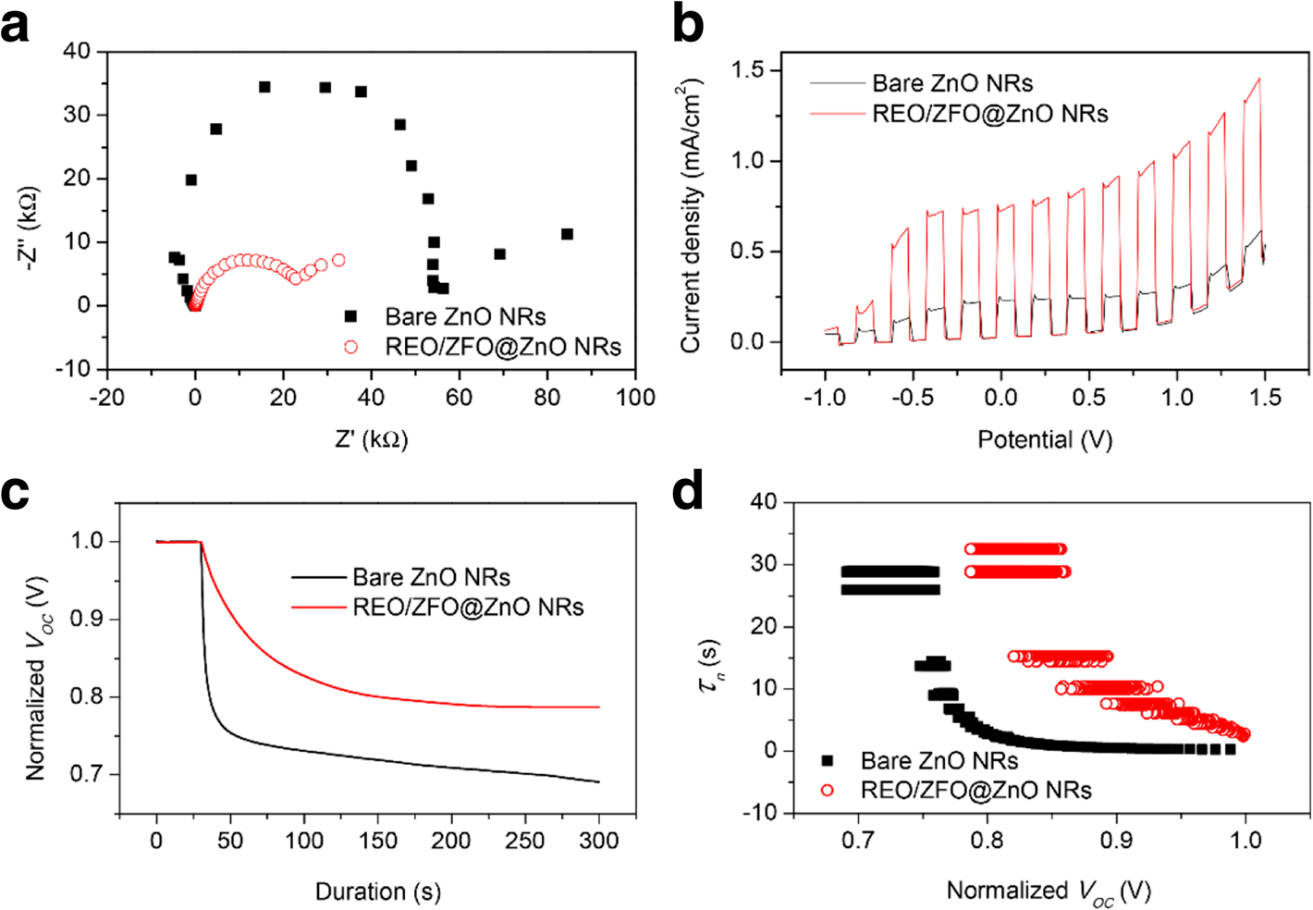
EIS plots (**a**) and chopped current density vs. potential (*J-E*) characteristics (**b**) of bare ZnO NRs (*black*) and REO/ZFO@ZnO NRs (*red*) under white light (AM 1.5G, 100 mW/cm^2^) illumination, (**c**) electron lifetime curves as a function of the normalized *V*_oc_ of the photoelectrodes made of bare ZnO NRs (*black*) and REO/ZFO@ZnO NRs (*red*), (**d**) OCVD spectra obtained from Fig. 6c

To further study the contributions of the REO/ZFO nanotapers to the overall PEC efficiency, electrochemical impedance spectroscopy (EIS), photocurrent-potential (*J*-*V*) and open-circuit voltage decay (OCVD) measurements are performed subsequently. EIS provides information about the interfacial properties of electrodes. The diameter of the semicircle in EIS correlates with the electron transfer resistance, reflecting the electron transfer kinetics of the redox probe at the electrode interface. Typical EIS plots of the bare ZnO NRs (black square) and REO/ZFO@ZnO NRs (red circle) in our PEC solution under illumination are shown in Fig. 8a. The EIS semicircle of REO/ZFO@ZnO NRs has a smaller diameter than that of the bare ZnO NRs, which indicates a faster charge transfer and a lower charge-recombination at the nanotaper/electrolyte interface due to enhanced light absorption and photo-conversion of ZFO. J-V measurements of the bare ZnO NRs (black) and REO/ZFO@ZnO NRs (red) are performed under white light illumination. Figure 8b shows that the REO/ZFO@ZnO NRs have an enhanced current density of 0.76 mA/cm^2^, which is higher than 0.24 mA/cm^2^ for bare ZnO NRs due to higher photo-conversion efficiency induced by enhanced light absorption and photo-conversion of ZFO, as well as the efficient electron-hole separation via cascade band alignment of ZFO and ZnO.

Subsequently, to understand the enhanced charge transport performance of our REO/ZFO@ZnO NRs with respect to the excited electron-hole recombination, open-circuit voltage decay (OCVD) measurements are performed. Figure 8c shows the OCVD curves of bare ZnO NRs (black) and REO/ZFO@ZnO NRs (red) as a function of time. These samples are illuminated for approximately 30 s to obtain a uniform open-circuit voltage (*V*_oc_), and the *V*_oc_ decay is then measured with the absence of illumination. Bare ZnO NRs show a faster *V*_oc_ decay due to the processes where holes accumulate on the surface of the ZnO NRs, and consequently, oxidation reactions involving the holes occur at the NRs/electrolyte interface. This promotes electron-hole recombination and the dissolution of ZnO. The REO/ZFO@ZnO NRs can then alleviate the *V*_oc_ decay as the photoelectron can migrate quickly with the aid of the nanotapers as pathways, leading to suppressed electron-hole recombination. Moreover, the passivation effect of the REO layer also slows down *V*_oc_ decay. Based on the *V*_oc_ decay rate, the electron lifetime curves can be deduced from the following equation [33]:

7 $$ {\tau}_n=-\frac{k_B T}{e}{\left(\ \frac{\mathrm{d}{V}_{\mathrm{oc}}}{\mathrm{d} t}\kern0.5em \right)}^{-1}, $$

where *k*_*B*_ *T* denotes the thermal energy, *e* denotes the positive elementary charge, and d*V*_oc_/d*t* denotes the derivative of the open-circuit voltage transient. Figure 8d shows the electron lifetimes *τ*_*n*_ as a function of the *V*_oc_ of the bare ZnO NRs (black) and REO/ZFO@ZnO NRs (red). The REO/ZFO@ZnO NRs exhibit a prolonged lifetime because of their cascade band alignment, branched structures, and passivation effect of REO layer, which effectively retard the recombination of electrons and holes.

**Fig. 9:**
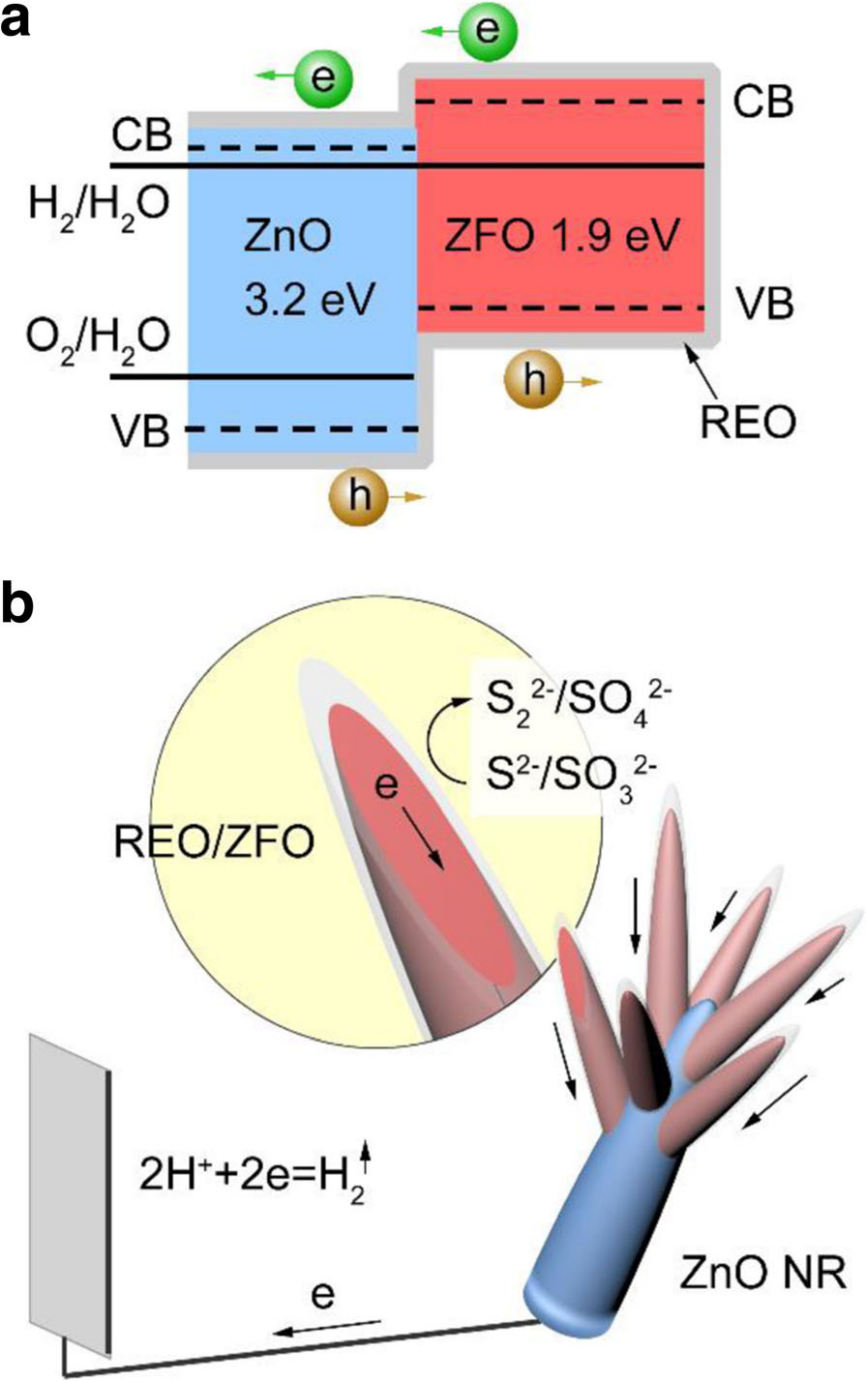
Band alignment (**a**) and PEC process (**b**) of the REO/ZFO@ZnO NRs

On account of the experimental analysis above, a schematic diagram concerning the cascade band alignment of ZFO and ZnO, and the PEC enhancement observed for REO/ZFO@ZnO NRs is presented in Fig. 9a and b, respectively. Many studies have been confirmed that ZnO and ZFO are n-type semiconductors with the electronic band gaps of 3.2 and 1.9 ~ 2.0 eV, respectively. Comparatively, their optical band gaps are little narrower. For example, Rekha Dom has revealed that the optical band gap of nano-sized ZFO has a range between 1.86 and 1.93 eV, depending on the fabrication methods [34]. So the little difference between the optical band gap and electronic band gap could be neglected for ZFO. Xuan Guo and Dongdong Qin have demonstrated that when ZnO and ZFO are coupled together, a type II band alignment is formed [35, 36]. The bottom of conduction band (CB) of ZFO is a little higher than that of ZnO for about 0.1 eV, and the top of valence band (VB) of ZFO is also higher than that of ZnO for about 1 eV [35]. After the band alignment is equilibrium, the Fermi levels of ZnO and ZFO will be unified. Therefore, under illumination, photo-generated electrons in the conduction band (CB) of ZFO are then injected into ZnO, which ultimately arrive at the Pt counter electrode to reduce the hydrogen ions to hydrogen gas (Fig. 9b). Most of the holes accumulated on the surface of the REO layers oxidize the S^2−^ ions to S_2_^2−^ ions. The role of the sacrificial reagent in the electrolyte (SO_3_^2−^) is to prevent a reverse reaction by reducing S_2_^2−^ to S^2−^ [37]. For the branched structures, the large surface area can absorb abundant light irradiation and supply plenty of interfacial area with electrolyte for PEC reaction [38]. Besides, a great number of nanotapers can act as numerous pathways to facilitate the charge transfer, leading to the enhanced PEC efficiency. Our previous studies have confirmed that the REO can also be used as the OH^-^ supplier to block hydrolysis of Na_2_S and Na_2_SO_3_ in the PEC electrolyte [23]. As a result, the PEC performance and the electron lifetime can be obviously enhanced by the REO/ZFO@ZnO NRs.

## Conclusions

Via recycling the REO slag as iron source, the branched ZFO nanotapers are fabricated on the ZnO NRs, and the resulting PEC anodes are demonstrated to be efficient for hydrogen generation. The PEC enhancement is attributed to the cascade band alignment of ZFO and ZnO and the branched structures. Moreover, the REO layer can tightly wrap around the ZFO nanotapers and act as a passivation layer, leading to improved electron lifetime. The products and the fabrication methods presented in the current study are highly suitable for developing various heteronanostructures for energy devices. Additionally, the REO/ZFO@ZnO NRs can increase the PEC efficiency dramatically compared with bare ZnO NRs, which demonstrates the potential applications of the REO/ZFO@ZnO NRs to future photoelectrodes. Overall, the products and the fabrication methods may also have a broad spectrum of applications in various nanotechnology fields, while facilitating the efficient use of REO slag.

## Abbreviations

IPCE: Photon-to-electron conversion efficiency
NRs: Nanorods
OCVD: Open-circuit voltage decay
REO: Rare-earth oxide
SEM: Scanning electron microscope
TEM: Transmission electron microscopy
XPS: X-ray photoelectron spectroscopy
XRD: X-ray diffraction
ZFO: ZnFe_2_O_4_
